# Disentangling Public Participation In Science and Biomedicine

**DOI:** 10.1186/gm525

**Published:** 2014-01-31

**Authors:** Christopher Kelty, Aaron Panofsky

**Affiliations:** 1 Institute for Society and Genetics, UCLA, 1320 Rolfe Hall, UCLA, Los Angeles, CA 90095, USA; 2 UCLA Department of Information Studies, GSE&IS Building, Box 951520, Los Angeles, CA 90095-1520, USA; 3 Department of Public Policy, Luskin School of Public Affairs, UCLA, 3250 Public Affairs Building, Box 951656, Los Angeles, CA 90095-1656, USA

## Abstract

**Background:**

This article provides a framework for disentangling the concept of participation, with emphasis on participation in genomic medicine. We have derived seven ‘dimensions’ of participation that are most frequently invoked in the extensive, heterogeneous literature on participation. To exemplify these dimensions, we use material from a database of 102 contemporary cases of participation, and focus here on cases specific to science and medicine. We describe the stakes of public participation in biomedical research, with a focus on genomic medicine and lay out the seven dimensions.

**Discussion:**

We single out five cases of participation that have particular relevance to the field of genomic medicine, we apply the seven dimensions to show how we can differentiate among forms of participation within this domain.

**Summary:**

We conclude with some provocations to researchers and some recommendations for taking variation in participation more seriously.

## Background

Public participation is a matter of growing importance in the biosciences, but we lack a comprehensive framework for making sense of the many forms that it takes. Is participation in a newborn-screening program the same thing as joining 23andMe? Is patient activism to steer research funding the same thing as a movement-run community health clinic? Is participation in a bone marrow registry the same thing as participation in a clinical trial? Is the active participation of parents in their autistic child’s therapy the same as the rejection of medical authority in the decision not to vaccinate? All of these things are labeled 'public participation' in biomedicine, yet they capture many different types of relationships between a lay public, doctors or researchers, governments and other stakeholders. The stakes of public participation for biomedicine are high, yet we lack an adequate language for distinguishing among the variety of entities, practices, and relationships that are designated as participatory and for evaluating their different effects.

This article provides a framework for disentangling the concept of participation. Based on the extensive and heterogeneous theoretical and practical literature, we have derived seven 'dimensions' of participation that are most frequently invoked to explain its function and goals. In order to exemplify these dimensions, we use material drawn from a database of 102 contemporary cases of participation - focusing for this article on those cases that are specific to science, technology and medicine. In this background section, we describe some of the stakes of the growing trend of public participation in biomedical research, with a particular focus on genomic medicine, lay out the seven dimensions and describe how our larger research project approaches the problem. In the discussion section we single out five cases of participation that have particular relevance to the field of genomic medicine, and we apply the framework to show how we can differentiate among forms of participation within this domain. In the summary we conclude with some provocations to researchers and some recommendations for taking variation in participation more seriously.

### Public participation in biomedicine

The participation of members of the public as research subjects and also as patients has long been a *sine qua non* of biomedical research. Yet, traditionally, scientists and medical researchers have preferred research subjects to remain 'subject' to their direction and control, not 'subjects' in the sense of people who 'talk back' to experts and insist on representing or pursuing their own interests. The reaction of the biomedical research establishment to such vocal and organized forms of public participation has ranged from paternalistic to hostile, seeing challenges to the authority of the establishment as attacks on the integrity of scientific research and medical practice. And thus the best known historical examples of this public participation - such as the women’s health movement, the Black Panther Party’s free medical clinics, and AIDS activism - have involved resistance to the biomedical establishment [1-4].

However, in recent decades public participation has lost much of its adversarial edge to become a key, legitimating idiom of institutions across the spectrum of society. Rather than asserting a separation from the public, government, civil society, and even business organizations now 'engage', 'solicit advice from', and 'consult with' the public in patterns of authority that emphasize shared responsibility over top-down government [5-10]. Sometimes these examples include grounded, practical attempts at participation, while in other cases they are more rhetorical or theoretical assertions. Conversely, cases of public exclusion, secrecy, and institutional autonomy are occasions for public suspicion and resistance. The biomedical research establishment has joined this trend to see public participation as good [11-16]. In the US, the NIH, long resistant to public 'interference', has come to acknowledge the role of disease advocates (for example, in attracting research support, developing drugs and therapies, and clinical trials) [17]. In France, the Muscular Dystrophy Association (AFM), has funded a substantial portion of genetics research [18]. And thus, even more than providing the idiom of legitimacy for contemporary institutions, public participation can at times fill in where the state (or market) is failing to supply crucial research goods.

In the past few years, scientific researchers have begun to take an even more active and creative approach to public participation, using the ubiquity of the Internet and its interactive affordances to solve a nexus of problems [19-24]. For example, biochemists studying protein folding have teamed up with computer scientists to build an online game, Fold It, that invites public users to compete in devising solutions to protein folding problems that are then used to 'train' computers to improve their protein structure algorithms [25-28]. The website Zooniverse is a platform where scientists studying problems as diverse as galaxy formation, bat vocalization, and cancer can recruit the public to aid in massive, labor-intensive data coding efforts [29-32]. Off-line, public health researchers have spearheaded participatory research as a means to recruit from hard-to-reach populations, prioritize communities’ own health concerns, and to develop interventions that rely on social capital and peer influence rather than top down messages or coercive rules to improve health [33].

The well known direct-to-consumer genetic testing company 23andMe has organized public participation to solve a knot of problems facing genetics researchers. By inviting users to pay (currently $99) for a SNP-chip-based genetic test and then answer online surveys about their health, traits, and ancestry, 23andMe has been able get about 400,000 people to pay to provide the company with valuable genetic and phenotypic data. The online interface allows 23andMe to stay connected with participants, introducing new studies and re-consenting them as necessary. 23andMe seems to be solving some of the ongoing cultural problems genetics has faced through fears of determinism, eugenics, privacy invasion, and discrimination. Its friendly interface, cultivation of well-informed participants, fun 'see for yourself' ethos, and multiple choice points for users to participate and disclose or not are all geared toward building senses of self-empowerment and trust and allaying fearful associations [34,35]. The traditional biomedical establishment has been duly skeptical of 23andMe’s participatory ethos, criticizing direct access to genetic data as dangerous to participants and potentially exploitative [36-41].

Thus, the stakes of public participation for biomedical research are high, both for these positive possibilities and for potential dangers (we need only think of vaccine skepticism and desperate patients’ pursuit of unvalidated treatments). But what is participation? Many different definitions have been offered, depending on the practical domain, or theoretical tradition. We begin with a definition from Bucchi and Neresini that was intended for the domain of science, technology and medicine: 'the diversified set of situations and activities, more or less spontaneous, organized and structured, whereby nonexperts become involved, and provide their own input to, agenda setting, decision-making, policy forming, and knowledge production processes regarding science (449)' [42]. A key feature of this definition - which is essential to our selection of case studies as well - is that participation involves influence of some broad public on a relatively closed institutional locus of power and decision making that is not exhausted by market, labor, or electoral relations. Thus, purchasing, working, or voting are not in themselves participatory but might be the point of departure for a participatory workplace, 'pro-sumption', or participatory democracy. The word 'participation' is often used in a very general sense to mean something more like the diversity or representativity of a population (as in 'participation in medical research by minorities') or the proportion of a defined population in reference to a defined activity (for example, 'voter participation' in an election), but these uses of the word do not rise to the level of participation we analyze here.

However, this basic definition of public participation conceals the variety of ways that participatory relationships can be organized. For example, Facebook is often identified as a participatory technology par excellence because it has over a billion users to whom it offers processes of communication and planning, information sharing, and interest promotion that were previously controlled by centralized media outlets. However, it looks much different as a participatory endeavor to other projects also labeled participatory, and we provide a framework here for understanding why.

### Disentangling participation

Our approach to disentangling participation is concerned both with the mechanics and structure of participation and with the way individuals talk about, experience or argue for or against it, both as a practice and as a theoretical problem. Our starting assumption is that there is a wide diversity of practices that get called participation, and that we need multiple cases from different domains in order to understand the nature of this variation. To accomplish this we employ two methods: one is an extensive investigation in the published literature, both contemporary and historical, to understand how the concept of participation has been handled in the past, which provides the warrant for our choice of dimensions. Existing frameworks [5,13,43-48] for understanding participation tend to be very domain-specific, from medicine to worker participation to art, but we have sought to distill commonalities across these multiple domains, in part to explore how much participation is or is not tied to specific grounded instances of it. As such, our reading of this literature was not confined to any particular discipline, and reveals that there are multiple non-overlapping places where participation has been explored at both a theoretical level and in terms of practical implementation. The most robust literature comes from the domains of 1) political theory and participatory democracy; 2) worker participation; 3) public administration and; 4) international development. Other domains such as art, architecture, urban planning, cooperative and socialist planning, management, participatory design and cultural studies - as well as approaches in medicine, healthcare and science - corroborate our choices. In the next section we discuss these seven dimensions in more detail, and describe how they relate to science and medicine.

**Figure 1:**
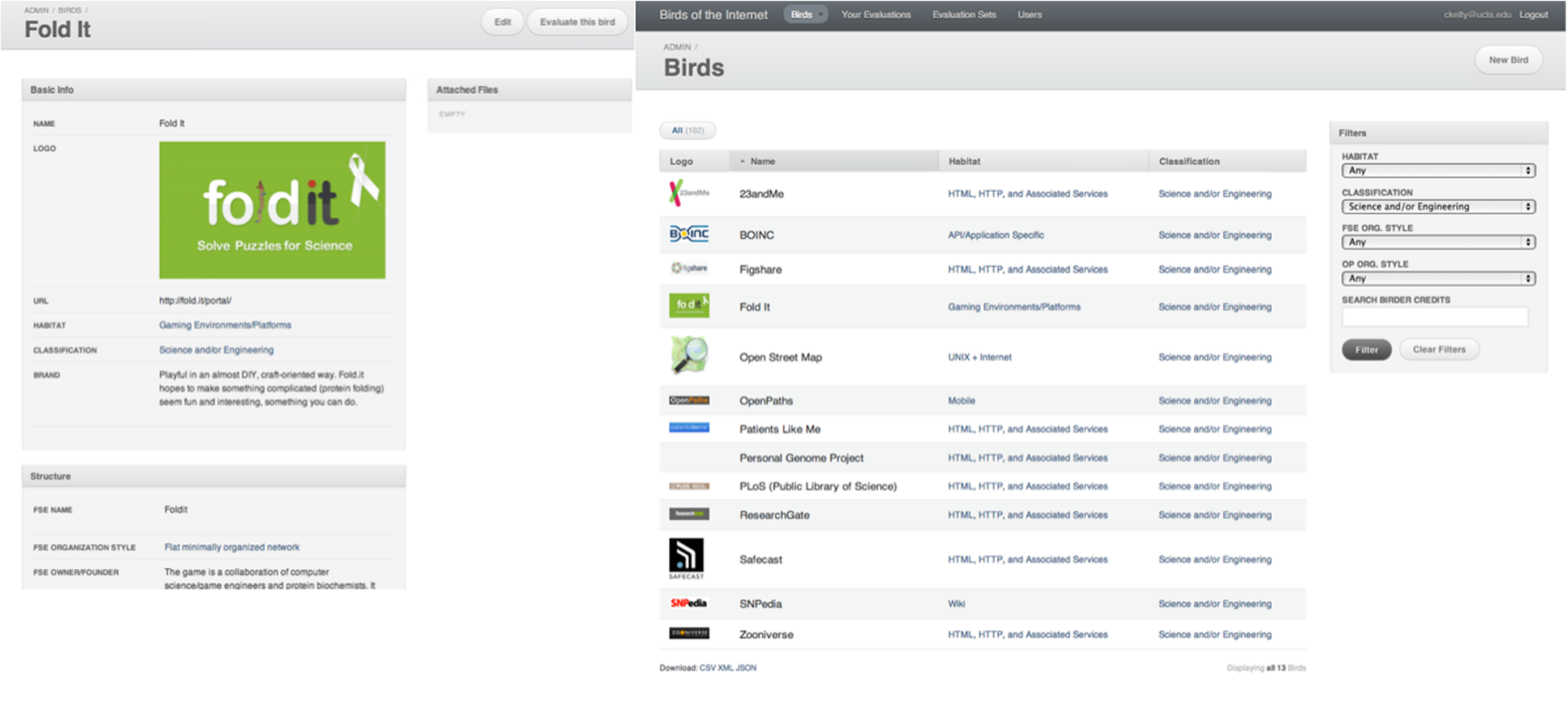
**Sample cases of participation.** Sample case study from the 'Comparative Analysis and Study Environment' (CASE) software framework. At left, the basic information for one case (9 of 32 fields shown); at right, a listing of available cases with selected criteria for filtering/sorting (13 of 102 cases shown).

The other aspect is the creation of an extensive database of structured case studies, conducted through ethnographic observation, interview and/or analysis of public documents. As of this writing, 102 case studies have been completed about instances of participation: groups, organizations, movements or projects organized in order to facilitate participation of some kind, and to benefit from it. The purpose of these case studies is more comparative than casuistic - we use the data collected to evaluate how participation is implemented in each case, and in order to exemplify different configurations of participation. Our approach is naturalistic in the sense that we choose any cases 'in the wild' that either explicitly claim to be participatory or are labeled as such by others. To date, our only limiting criterion for this set of cases was that they all involve the use of the Internet or other information and communication technologies. Each case study answers 32 structured questions (described in [49]), and is based on public records, websites, news articles, discussions, and interviews with key participants. The cases were drawn from a range of domains: Free/Open Source Software (FOSS) (19); social networking (14); science/engineering (14); culture industry/other (13); activism (10); education (7); citizen journalism (7); social entrepreneurialism (6); craft/DIY/consumer goods (5); games/persistent worlds (4); and forum/mailing list (4). Figure 1 provides an example and see [50] for more details.

**Figure 2:**
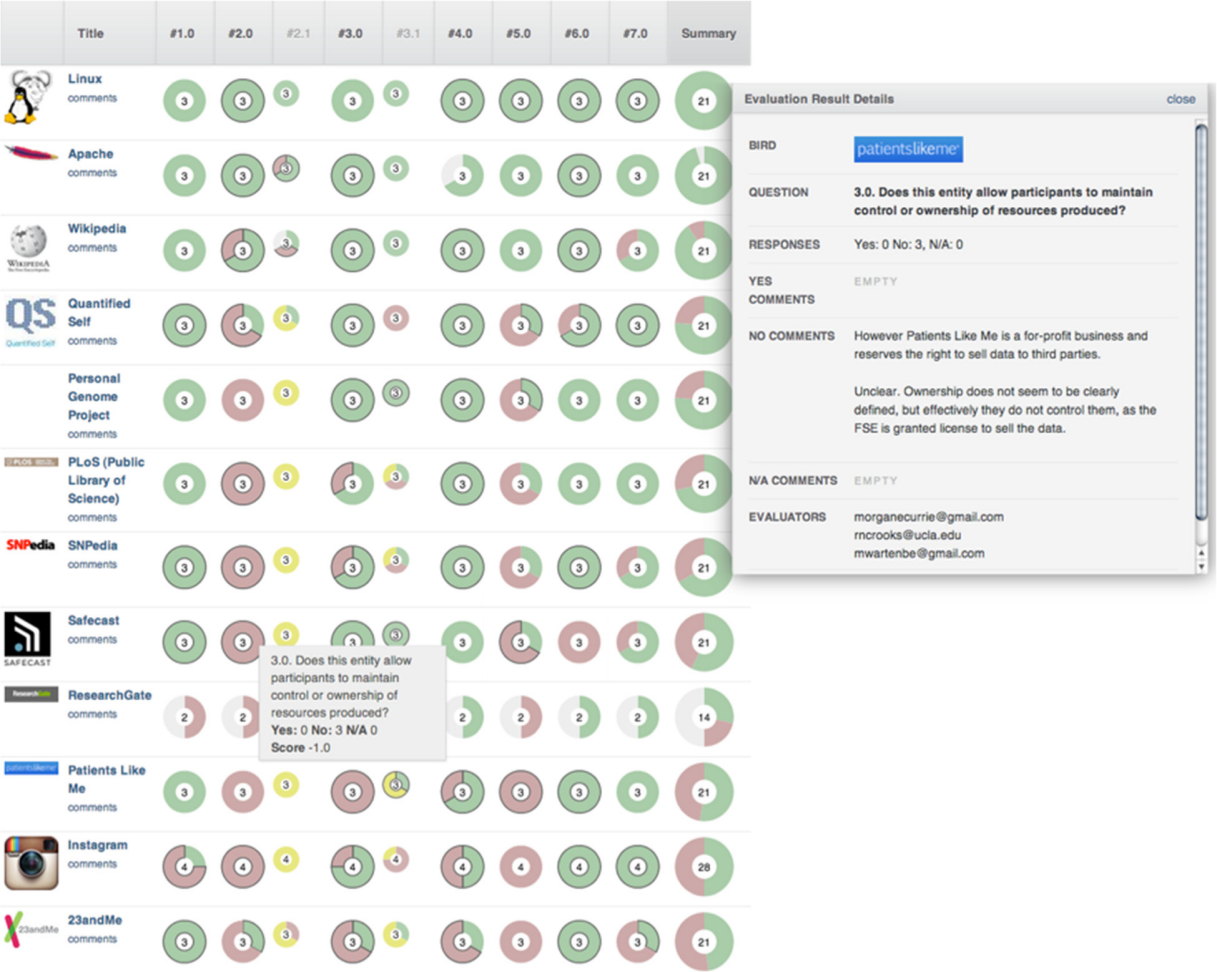
**Sample evaluation grid.** Sample evaluation grid from the 'Comparative Analysis and Study Environment' (CASE) software framework. For each question 1 through 7 the circles display researchers’ collective consensus score (+/−) of each case (for example, Q3 concerns control and ownership of resources). In the center of each circle is the total number of evaluators; the pie chart shows the degree of consensus - all positive (green), mixed and all negative (red) (yellow indicates a sub-question that is not applicable); a black outline represents interpretations or comments added by the evaluator. Cases can be re-sorted by question (Q2.1 and Q3.1 are sub-questions).

These two approaches were combined by using the seven dimensions to evaluate each of the 102 cases (6 to 7 researchers evaluated a subset of the cases, resulting in 3 to 4 evaluations for each case). By doing so we generate a 'consensus score' for each case that represents the expert analysis of our team about the degree to which each case includes or excludes each of the dimensions. Each case therefore has a seven-dimensional 'signature' that we can use to compare the cases and to look for patterns that represent different modes of participation (see Figure 2 for an example). In terms of other methodological approaches, ours shares some similarity with so-called 'grounded theory' and 'fuzzy-set' social science [51-53]. However, our intention in this paper is primarily descriptive and aimed at an empirical specification of the meanings of participation; we suggest some future directions for research in the summary.

#### Educative dividend

Perhaps the most commonly remarked upon, and most important aspect of participation is its educative potential. Education here can mean both the simple acquisition of knowledge and skills, but also the knowledge of how to participate better - civic virtue. The cultivation of civic virtue has a grand tradition, including the works of Machiavelli, Locke, Rousseau, Mill, De Tocqueville, the 19th century French socialists like Proudhon and Fourier, and the 20th century British socialists like GDH Cole. Civic virtue is necessary in order for participation as a collective effort to benefit everyone, but it is also clearly difficult to instill such virtues in (all) individuals in the first place. Many such theories, therefore, rely on the idea that participation itself leads to greater civic virtue: that one learns to participate by participating. Carole Pateman’s 1976 classic *Participation and Democratic Theory* explores the history of this theory; according to Pateman, democratic theories of the early 20th century abandoned a participatory theory because the sociological evidence concerning engagement and apathy led them to assert that most people are constitutionally incapable of, or simply uninterested in, participating. Participatory theory, by contrast, offers a different explanation - that apathy or lack of engagement follow from a lack of participation, rather than preceding it. If there were more opportunities for participation, individuals would cultivate those skills to a greater extent and ultimately develop civic virtue and participate to a greater extent. Pateman suggests that the 'patterns of authority' across society (at work, at home and locally as well as nationally) have an effect on this cultivation [54-56].

In science and medicine, education is very often a key good presumed to result from participation: whether that be knowledge of self or knowledge about the world, more participation reduces the ‘knowledge’ deficit of citizens often decried in discussions of scientific literacy [57,58]. Although it should be clear from both the experience of practitioners and the curricular expectations that 'learning by doing' is key to becoming a better scientist, it is rare to hear it suggested that non-experts should actually be encouraged to enter science this way. 'DIY' science and some forms of citizen science are therefore often met with concern for exactly this reason [59,60].

#### Goals versus tasks

Participation in decision-making is among the most intuitive senses of what it means to effectively participate in something: the possibility of affecting the setting of goals not merely the accomplishment of tasks oriented towards those goals. From worker participation to participation in development planning to participatory democracy, the move has always been ‘upward’ towards increasing involvement in the management, agenda and operations of any project or organization [43-45]. At the same time, bureaucracy and the division of labor provide a countervailing ‘downward’ force that has worked to manage scale, equity, and the distribution of power, often restricting the sense of participation [61-64]. Classic labor analyses (both Marxist and non) have noted the disempowering aspects of this distinction [65,66].

A key aspect of this dimension therefore has to do with the structuring of participation, and the development of affordances and metrics (see the 'Metrics of participation' section below) that enable participation, make it easier to do, or provide feedback on the effects of participation. If participation is too highly 'engineered', it is likely to seem inauthentic and the goals will appear inaccessible to the participant. Critiques of participation in international development [67] and science studies [68] often point out that when participation is too bureaucratized it generates 'professional' participants who know how to work the system (but lack ‘civic virtue’). However, it is also possible for participation to appear too open and unstructured, leaving no certainty about how to participate, or relying on extensive pre-existing experience or knowledge in order to do so, as is often the case in free software projects.

In science and medicine, decision-making can refer to multiple domains: in medicine, participation in the decision-making between a doctor and patient [12,15,69] or participation in the design of a clinical trial or research agenda [18,70,71]; in science it can mean participation in the design of research, or involvement in the general societal goals and impacts of research. Even strictly amongst scientific researchers, the participation in 'goals' is often the province of only those most advanced in their research careers - and it is a mark of prestige to become the researcher who can set the agenda for research. Much research has recently investigated how agendas are set in response to social and political concerns [72-75].

#### Control and ownership of resources

A third and key dimension of participation is the definition and control of resources produced. 'Resource' has a deliberately tangible sound to it, but many cases of participation may not produce anything tangible or circulatable. Building a barn, voting on a government, or creating a local budget all imply different meanings of the term 'resource' and in each case its production, distribution and disposition will differ. In many contemporary cases, it is intellectual property and contract law that govern the resources in question, and so there is often a concrete legal locus to the resources produced by participation [76,77].

Additionally, more than one resource may be at stake in any given case of participation. One such resource might, for example, be the educative dividend described above - tangible acquisition of skills by an individual [78] - while for the entity enabling it the resource is data, information or materials donated by the participant. Clinical trials are a clear example of this duality: health, treatment or medicine is the resource for the patient, but data and information comprise the resource for the researcher [70,71]. Identifying the resource is a necessary step preliminary to defining how it is made available - individually, collectively or in some other arrangement.

Contemporary participation in the 'information age' is clearly different from prior experience, insofar as information production is a much more common activity across all domains, and such information has been given value, often monetary value, in new and experimental forms - one need only consider the conflicted story of gene patents for instance. Nonetheless, it helps to think of 'resources' generally in this context because information and information resources are neither new, nor is their value - but the scale, mode of circulation and 'tangibility' (or ‘objectivity’ perhaps) of such resources are no doubt taking new and different forms.

#### Exit…

A classic in economic analysis, Albert O Hirschmann’s *Exit, Voice and Loyalty* explored a theory of loyalty in response to decline in organizations by asking when consumers (or voters) choose to respond with exit and when they choose voice [79]. Exit refers to the act of switching to a competitor (as in the case of competing products), or dropping out of a market or a political sphere altogether. Hirschman’s presentation of exit and voice is not explicitly figured as a theory of participation, but as one of loyalty. It contains, however, a key feature of participation rarely remarked on - its character as a voluntary activity. Almost no situations outright prevent exit (indentured labor contracts or slavery might be such cases), but many can make it difficult, costly, dangerous or threatening to do so (as in the case of clientelism in politics, or perhaps a loss of health effects by failing to complete a clinical trial). Loss of reputation, social ties and potentially the threat of retribution can all be adverse correlates with the value of participation. And key to such concerns in the contemporary era is, again, the disposition of resources, that is to say, 'can you take them with you' if you exit?

Exit, conceived in terms of medical or scientific activity, raises complicated issues about paternalism, freedom of choice and responsibility. Patients in clinical trial treatment regimens are often not at liberty to exit without some kind of loss (access to medicine and medical treatment principally), even though it is a principle of human subjects protection that individuals be guaranteed the right to cease participation at any time. Participation in scientific research might be suitably voluntary, but exit might still come with penalties - loss of data, loss of access to a network, or other such resources produced through the act of participation.

#### … and Voice

Hirschman’s alternative - 'voice' - was meant to capture the tension between loyalty and defection [79]. What were the conditions, he asked, in which a consumer or voter was moved to complain (or campaign) rather than switch? Indeed, voice is generally treated as one of the most, if not the most, central capacities for participation in democracy - from the Greek Agora to public coffee house and the New England town hall meeting [80,81]. The expansion of voice by the Internet is often given as one reason for its 'democratic' nature; conversely, it is also suspected of producing echo chambers and 'bubbles' that limit the effects of participation [82,83].

From the perspective of a theory of participation, however, it is the effectiveness of these forms that matters: does feedback register, and if so how? Is it akin to participation in goals, not just in tasks? And are participants able to monitor when their feedback is being heard and acted upon? Is it structured in such a way to be influential or merely to provide the appearance of influence? Additionally, for voice to be effective, there can be no adverse effects from speaking up, no punitive effects or retribution for doing so.

Patient advocacy groups would appear to be the most obvious form of voice in recent history, from AIDS activism to those arguing for access to medicine, or research on neglected diseases to the activists in the vaccine and autism controversy, a key reason to participate in many different kinds of public projects is to 'make your voice heard' whether conceived of as the exercise of voice in a public sphere, or participation in interest group politics.

#### Metrics of participation

A key aspect of theories of participation is a consistent concern with the collective (rather than merely the individual) experience of participation (hence the adjective ‘civic’ rather than personal or private virtue). But rarely do these theories explain how individuals can witness or understand the effect of participation. Metrics or signs of participation can model the outcome of increased participation, or allow an individual to monitor (to varying degrees of detail), his or her contribution to something, and its effects. Such metrics can range from the simple tally of votes to more complex accounting and audit of individual participation, or run towards more qualitative feedback and interaction, as in the classical forms of apprenticeship or mentoring. The rise of statistics and polling as technologies of collective representation has played and continues to play a central role in making such participation visible [84-90]. Among all the dimensions of participation, this is perhaps the least often to be explicitly named, but the most likely to produce the experience of linking the individual and the collective experience together in meaningful ways.

It must also be noted that, in the age of 'big data', it makes a difference which metrics are available to participants and when; every computer-enabled service keeps data of some kind, and large corporations like Facebook or Google keep massive amounts - but very little of it is accessible outside of the corporation, sometimes for strategic reasons, sometimes due to concerns about privacy, and sometimes simply because it is not perceived as relevant or meaningful data. Metrics meaningful to participation are evidently those that participants can see, and perhaps even manipulate. Medical data are obviously of a different order than other kinds of data - hemmed in by regulations and concerns about privacy, public health and individual safety. 'Metrics' then are often of a kind that represent general trends, rather than revealing information about individuals. Nonetheless, it is often necessary to provide such feedback in the service of producing an experience that is collective in nature, rather than individual.

#### Subjective, communicative experience of participation

Lastly, participation is fundamentally a collective experience amongst individuals - and often participation is understood as a convivial, face-to-face and affective experience amongst peers - as opposed to anonymous, disconnected or rationalized intercourse amongst strangers (for example, in a market). As a result, many theories of participation assume that individuals will have or will develop a method of experiencing participation as a collective experience, somewhat like Durkheim’s notion of 'collective effervescence' [91]. This includes a capacity for communication amongst members of the organized public as they participate, and perhaps also an affective language or relationship that develops - commitment, frustration, anger, pleasure, satisfaction. Restricting or deliberately severing such ties has the effect of making the experience seem less participatory, even if the functional outcome is the same. Theories of participation often reference these affective or communicational aspects without necessarily making them central to a theory [92,93].

Table 1 summarizes these seven dimensions.

**Table 1:** Summary of seven dimensions of participation

**Dimensions**	**Description**	**Representative scholarly literature**	**Contemporary cases that exemplify the participatory dimension**	
			**Strong**	**Weak**
1. Educative dividend	Learning something valuable, especially learning how to participate effectively	[54,78,94]	Zooniverse, 23andMe	Match.com, BOINC
2. Goals and tasks	Participants not only undertake tasks but help set goals	[15,43,44,67,69,95]	Linux/Linux Foundation; PXE International	Pinterest; current TV; Patients Like Me
3. Resource control	Participants get to control (own or use) resources, not merely produce them	[65,96-99]	Second life; Mukurtu; SNPpedia	Patients Like Me
4. Exit	Capacity to leave without penalty and with resources	[79]	Global Voices; SNPpedia	Facebook
5. Voice	Opportunities to 'speak back' in order to influence outcomes	[79,80]	Wikipedia; Apache	OKCupid
6. Visible metrics	Empirical demonstrations of the connection between participation and outcomes	[56,85,90]	Foldit; 23andMe	Revision 3
7. Affective/communicative capacity	Participants have opportunities to communicate amongst themselves to produce affect, affiliation, and sociability	[91-93]	Instagram; PXE international; Patients Like Me	Bitcoin

Summary of seven dimensions of participation.

## Discussion

**Figure 3:**
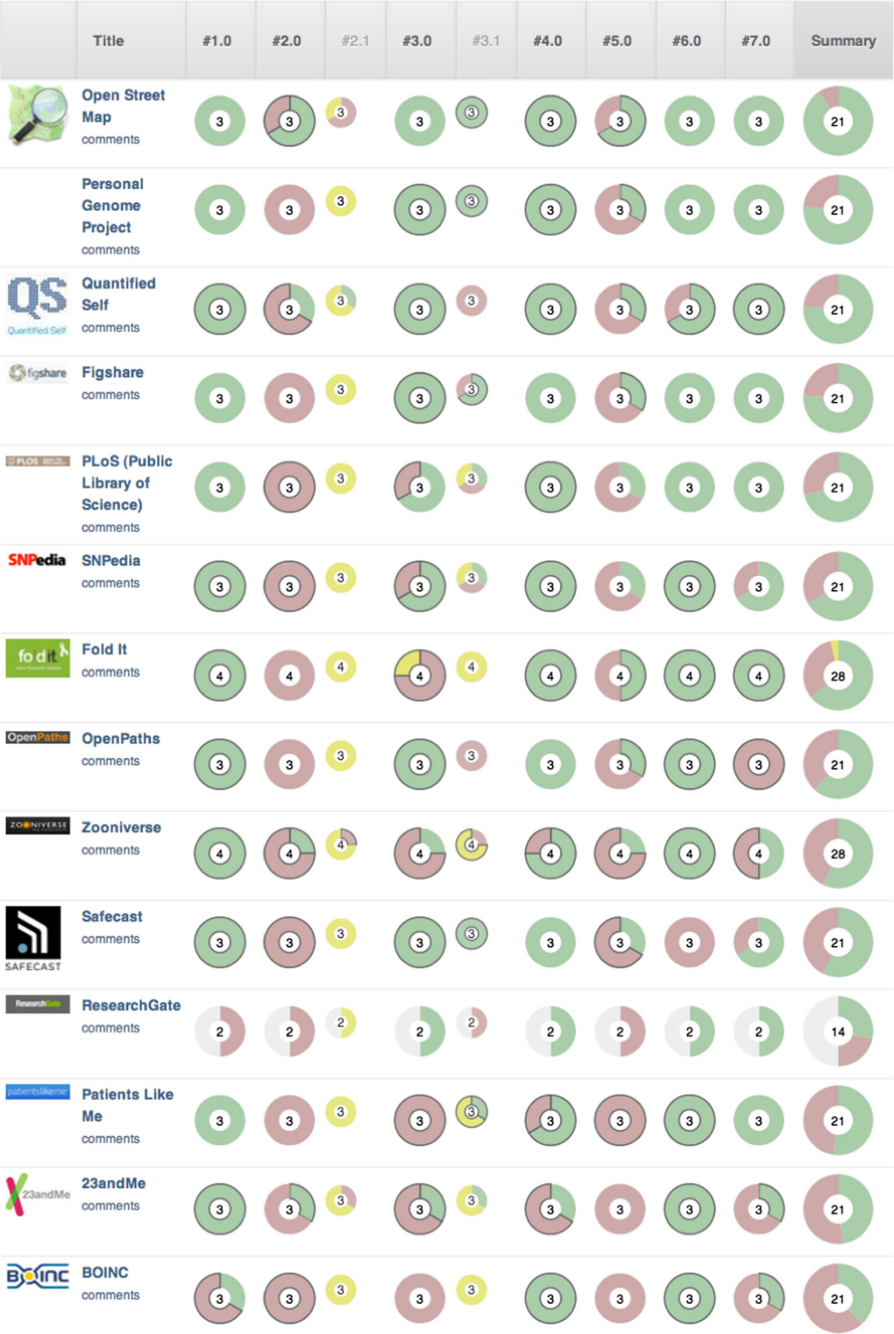
**Results for science/engineering/medicine cases.** Fourteen cases of participation in the domain of science engineering and medicine. Question numbers correspond to the domains in the 'Background' section; cases are ordered by the total evaluation ('summary') at right.

Any given case of participation can combine these seven dimensions in different degrees - some will focus on education and voice, for instance, others on metrics and an experience of collectivity. We have evaluated our 102 cases in order to determine what distribution of dimensions occur across cases of widely different domains - from free software and citizen journalism to science and medicine to economic participation. Figure 3 summarizes the evaluation of 14 of these cases that are categorized as being in the domain of science or medicine in our database. From even this subset, it is clear that there is much diversity in the presence or absence of the seven dimensions of participation listed here. Nearly all of the cases have a strong commitment to education (dimension 1 in Table 1), but almost none of them seriously consider allowing participants to be involved in agenda setting (dimension 2). Control over resources (dimension 3) varies greatly, as does the relationship between exit (dimension 4) and voice (dimension 5). But almost all are committed to providing good metrics of participation, and about half are devoted to facilitating communication or affective bonds between participants. These 14 cases were selected here to exemplify the domain of science and medicine, but the same diversity exists in the larger set of 102 cases.

In order to describe in more detail what these findings mean, we here examine several cases in more detail, including two not in our database (because they precede the advent of the Internet) but relevant to the issue of science and medicine. The goal is to exemplify how these cases combine different dimensions of participation; in the summary we propose some questions for thought and future directions based on these evaluations.

### AIDS activism (mid-1980s to mid-1990s)

Participation in medicine arguably received a huge boost in the 1980s with the advent of AIDS and the activists who engaged with the medical establishment. Although this case is more clearly understood in the literature as a case of a social movement [3,100], it inaugurated a wave of patient advocacy and demands for greater inclusion [70,71,101] that has continued into the present day. These concerns now structure approaches to access to medicine, clinical trials, and agenda-setting in research [75,102]. AIDS activism in the 1980s exemplifies several of the dimensions of participation laid out here. By becoming engaged, AIDS activists educated themselves about everything from the process of science to the biology of AIDS, and through that participation learned how to engage the medical establishment in order to participate in the setting of goals as well as tasks. Although the struggle could be conceived as one of resources, the concern was less with direct control and more with influence over the outcomes of decision-making. Exit was eschewed in favor of voice - speaking out and protesting. But perhaps most significantly, the experience of AIDS activism made gay activists visible to themselves and to others in a new way, and created a collective experience that defined a generation, created lasting bonds, and created new professional and personal possibilities.

### PXE International (1995 to now)

PXE International, a patient advocacy group representing those with pseudoxanthoma elasticum (a rare genetic disorder that mineralizes elastic tissues in certain parts of the body), was founded in 1995 by Sharon and Patrick Terry because of their frustration with the pace and disorganization of research into PXE, which afflicted their children [103]. PXE International is well known for its active role in initiating and organizing research, not just organizing patients and advocating on their behalf or funding scientists at a distance [104]. Though lacking science training, the Terrys participated actively in the laboratory research that would lead to the discovery of the mutation responsible for PXE, and the scientists they worked with agreed to assign rights to the gene patent to the organization. PXE International has served as a model for other advocacy groups seeking to form themselves as research organizations [18,105,106].

We can see that PXE International includes many, if not all, of the dimensions of participation. Disease sufferers, and especially those with rare diseases, often suffer from social isolation and alienation in addition to physical disease effects. Advocacy organizations help give disenfranchised individuals voice and they connect them into networks of affect and mutual support. PXE International has had a trebly effective educative dividend: patient participants learn about PXE and how to deal with it, scientific knowledge about PXE has expanded through the organization’s activities, and participants - both scientists and advocates - have learned how to engage each other, to participate together, more effectively. The organization has made part of its mission educating other organizations how to replicate their successes. Unlike the traditional advocacy model where lay people participate in research by being research subjects and having limited effect on the goals of research other than by sponsoring certain projects (or asking politicians to sponsor them), PXE International members have directly interacted with scientists and work in partnership with them to decide which research directions to pursue. Further, their control of resources is substantial: beyond the patent to the ABCC6 mutations causing the disease, PXE International has built and maintained an important biobank of patient samples and data, and they use these to attract researchers, enforce cooperative relations among them, and steer the course of their work. PXE International as a collective entity is nearly an ideal type of the participatory organization by our dimensions. While this is true at the collective/organizational level, we often think of participation in individual terms, and the experience of the average individual participant may not be the same as this collective story of success.

### Patients Like Me (2004 to now)

Patients Like Me provides a particularly striking case of participation in medicine enabled by the Internet and social media. Begun in 2004, the website is devoted to bringing patients together outside of conventional channels of either formal medicine or support groups and patient organizations. Patients can sign up and share information about their condition with others, including extremely detailed daily or hourly reports on treatment, course and outcome of a disease. Everything from mood and interests to drugs and treatments taken, to side effects and related conditions can and often are reported. Patients can message each other, 'follow' each other and offer support in ways that are similar to other social media sites [107].

Patients Like Me clearly has strong educative potential, effectively uses metrics to display individual and collective participation, and promotes strong affective and communicational bonds amongst users. The value for patients is clearly in the creation of new relationships, knowledge sharing and learning about treatment and the experience of a disease. Patients get out of it what they put in, and have access to a potentially much larger pool of people in similar situations than they might otherwise. The site is also very metric-driven: one can see not only one’s own progress and change over time, but that of others with similar conditions. The use of metrics can be a very important feature in making visible the effects of participation to all those involved, and Patients Like Me is built on the visibility and comparability of such metrics.

But on the flip side, the project does not facilitate good resource control and participation in agenda-setting. Patients Like Me makes money by selling the data that patients provide - anonymized and tailored for different clients, and they are honest about this aspect of the business. But controlling the data one provides is not something patients can do - either they give up control or they must exit. Similarly, there is no direct channel for patients to be involved in decision-making, either about their own data, the general decision about the choice of clients, or marketing of the available data.

### 23andMe (2007 to now)

Though direct to consumer genetics is a growing sector, 23andMe is the best-known and most fully realized of the efforts. For a small fee (currently $99), people can send in a DNA sample that is sequenced with a single nucleotide polymorphism (SNP) array. The resulting data are presented in terms of the individual’s disease risks, trait propensities, and biogeographic ancestry. Social networking functions allow individuals to communicate their traits with others and also to connect with genetically identified possible relatives in the database. Beyond the broader fact of gaining access to heretofore exclusive information, the main participatory element is that users are asked to fill out numerous surveys about themselves. The information is used partially to customize the individual’s risk profiles, but it is also the basis of 23andMe’s research agenda, which has published research on many physical traits, population genetics, and Parkinson’s disease [35,108-110].

On November 25, 2013 the FDA told 23andMe to stop selling genetic tests arguing that they comprised a medical device providing medical interpretations that had not been validated and approved by regulators. This action reflects, in part, a conflict about public participation in science. 23andMe wants to encourage participation in the world of biomedical information; give people information and let them do what they want. The FDA, in contrast, is asserting a more traditional elite-driven model; information must be controlled and validated by experts before the public may have access to it. 23andMe is implicitly in the business of 'disrupting' the existing regime, but its authority must still be respected.

The educative dividend of participating in 23andMe is clear; participants learn about genetics in general and their own genetic background in particular (though how an individual should interpret these data is ambiguous). There are also clear metrics of participation - the site reminds users how many surveys they have filled out and prompts them to fill out additional ones. However, participation in the goals is limited. Occasionally users are polled on which research project 23andMe should pursue, but more robust agenda setting is denied. Users are allowed to access their risk profiles and download their genetic data, but by agreeing to participate users grant 23andMe ownership, control, and discretion about sharing the genetic and survey data even after a participant leaves. User participation is governed by an institutional review board, and there is a procedure for withdrawing, but it is unclear what happens to data that have been shared extramurally with research partners. Thus, the option of exit is ambiguous. Finally, via social networking functions, 23andMe allows some affective connection and communication - notably among previously unknown 'relatives'. Communication among users and talking back to the organization are features facilitated by the site’s blog and comment system.

At first glance, these affordances (entirely typical among such websites) do not seem to be structured to enable much voice for users about the conduct of the organization. Yet, when 23andMe announced a patent for genetic variations associated with Parkinson’s disease, these became the venue of a heated protest by participants. Two points are relevant here. First, that even a participatory dimension whose affordances do not appear that radical (comment fields are ubiquitous) can still enable participant revolt. Second, the controversy overall reflects conflict over the 'ownership and control' dimension discussed above. For protestors, the patent belied the company’s ethos of openness and sharing. Where their participation had been portrayed as a research partnership, suddenly 23andMe’s commercial character came to the fore and participants were revealed to be (in part) resources to be mined.

### SNPedia (2006 to now)

SNPedia is an online wiki that allows users to interpret their own genetic SNP data using publicly available associations with particular SNPs [111]. The site is partnered with a piece of software called Promethease that connects an individual’s SNP data to the wiki. Whereas 23andMe offers users a processed version of this information, using proprietary algorithms to weight research findings and produce a personalized risk estimate, SNPedia provides do-it-yourself users the raw information. This information is more varied, voluminous, and detailed; it offers users the opportunity to make their own evaluations about risk but also to pursue information about the actual molecular biology in the published literature that may be behind a given SNP association. In addition to using the resource to interpret their own genetic data, users can participate by updating the wiki (linking SNPs to published findings and offering their own annotations). SNPedia contrasts with 23andMe and the Personal Genome Project in that it does not ask for users’ DNA information for its own purposes; it is only a resource by which users can interpret their own data. It does have an implicit relationship with those sites, however, because SNPedia does not offer users a way to sequence their data, they must bring their own, and 23andMe and PGP are two common sources [112].

Once potential users overcome the significant barrier of acquiring their genetic data, they can reap a significant educational dividend from using SNPedia. The information is not for casual users in that they have to be willing to interpret and explore information that is much closer to the scientific literature and less standardized than 23andMe, for example. Yet they have the opportunity to explore more of the current state of the art. Since SNPedia has few goals and tasks outside the resource itself, there is relatively little opportunity to participate in this regard. There are no metrics of participation apart from the links between genetic data and the information in the wiki. And opportunities to speak back to the entity and affect its course are primarily achieved by sending emails to the project’s leader, Michael Cariaso. Thus, voice and goal influence are largely informal, and occur at his discretion. Under a Creative Commons license, users have access to the resource without paying and they maintain control of their genetic data without ceding it to the organization. Thus, participants have full capacity for exit. In contrast to other entities where full participation must be controlled for the purposes of pursuing profit or an autonomous research agenda, in SNPedia there is very full participation along certain dimensions and those dimensions where it is lacking are due to the limited ambitions and informality of the enterprise, not active efforts at control.

## Conclusion/summary

The cases presented above are intended to exemplify the different dimensions of participation, and to demonstrate how different kinds of participation can be at stake in different projects. On the basis of this diversity, it is possible to conclude that different styles or modes of participation emphasize or de-emphasize different aspects, and that an empirical analysis might reveal the distribution of these styles of participation and potentially their efficacy or outcomes as well. At an empirical level one can ask what the 'signature' of participation is by looking at more than just one dimension. From this perspective, the relatively narrow concern with patient participation in treatment decisions concerns only a single dimension, whereas a case like PXE engages all of them. This is an implicitly normative claim, to be sure, but it also points the way towards understanding distinct advantages or disadvantages that might be associated with one or another signature of participation.

Indeed, there are multiple ways to both succeed and to fail at participation - and when it does not work, it is not necessarily 'participation' in an abstract sense that fails. Does participation need to have all seven dimensions? Which signatures are good for what reasons? Good for improving individual and public health? Good for introducing efficiency or profitability into healthcare or scientific research? Good for innovation or discovery? These are questions that cannot be assessed without a more robust model or theory of participation. On the basis of these dimensions we can hypothesize the mode of participation that results from the emphasis on one or another dimension. Facebook, for instance, emphasizes the collective experience and the use of metrics to display the social network to its users, but deemphasizes control over resources or access to goals. As such it can be labeled a more 'extractive' form of participation, focused on transforming resources valuable to one group (users) into resources valuable to another (advertisers). Conversely, projects like Wikipedia, which emphasize the first five dimensions, but are less interested in displaying or facilitating a feeling of participation, are more 'volatile' and unstable - committed to openness but struggling with sustainability; open to all comers, but not necessarily 'user-friendly'. In the domain of genomics, 23andMe clearly looks more like the former, and SNPedia like the latter (and this is no historical accident).

Similarly, a case like PXE, with its emphasis on nearly all the dimensions, is less intensely focused on the individual’s own responsibility for (and freedom to) monitor and understand his or her own health - and more oriented towards the production of community and collective control and power. When participation is understood primarily as a form of consumption (giving individuals data about themselves but unconnected to any other patients or collectives), then its signature is of one type, and with the framework above, it might be possible to both identify such instances, and ask 'what choices about the design or management of a project can shift it more in the direction of one signature than another?'

With scientific institutions being more exposed to public scrutiny and bearing more of a burden to justify themselves not just to authorities but to the citizenry that supports them, accommodating public participation will become more of an obligation for scientists. This is an emerging obligation that carries considerable risks in terms of loss of control and rise of contention in the research process. Yet it also offers many intriguing possibilities for new forms of collaboration, data, knowledge production, funding, and serving public needs.

So what lessons can a clinical scientist, genomics researcher or entrepreneur draw from this analysis? As we have shown, public participation is much more complicated and multidimensional than it is usually taken it to be. It is more than simply enrolling participants and telling them what to do; at least, participants today tend to expect much more than that. When seeking to engage public participation in their research, scientists would do well to think about the dimensions we have identified and consider the synergies and trade-offs among them. Scientists would seem to be most comfortable with the educative, task, metrical, and community dimensions of participation. But participants themselves often want more. They want influence over goals, they want to share in the benefits of the resources created, they want genuine opportunities to engage scientists without too many barriers of expertise and authority. These are among the lessons of the brief case studies presented above. What scientists might realize is that scientific authority and knowledge production are not necessarily zero sum games. As the PXE International example and many other cases of patient advocacy in genetic research show, close participation can produce synergies and energies that catapult basic and clinical science forward [106]. New productive equilibria can be reached that are superior to whatever degree of authority scientists cede to participants.

Finally, it has long been a key feature of the literature on participation that participation is inherently political - meaning that it is about the redistribution of resources and the struggle for power over decision-making. But it is also about collective experience and the feeling of being a part of something larger. While incorporating strong forms of participation may therefore come with risks - that doctors might have less claim to expertise, or that companies might have less control over the circulation of data - it also comes with benefits that far exceed the immediate domain of patient care or research: it can create patterns of authority and responsibility that influence other aspects of social and political life beyond that of medicine.

## Competing interests

The authors declare that they have no competing interests.

## Authors’ contributions

CK contributed to the case study research and co-wrote the article; AP contributed to the case study research and co-wrote the article. Both authors have read and approved the article.

## Authors’ information

CK is Associate Professor of Anthropology and Information Studies in the Institute for Society and Genetics at UCLA. AP is Assistant Professor of Public Policy in the Institute for Society and Genetics at UCLA. Together they oversee an NSF-funded project on participation in science and medicine (information is at http://recursivepublic.net/).
